# The Principles of Nerve Cell Communication

**Published:** 1997

The nerve cell, or neuron, is the key player in the activity of the nervous system. It conveys information both electrically and chemically. Within the neuron itself, information is passed along through the movement of an electrical charge (i.e., impulse). The neuron has three main components: (1) the dendrites, thin fibers that extend from the cell in branched tendrils to receive information from other neurons; (2) the cell body, which carries out most of the neuron’s basic cellular functioning; and (3) the axon, a long, thin fiber that carries nerve impulses to other neurons.

Nerve signals often travel over long distances in the body. For example, if you step barefooted on a sharp object, the sensory information is relayed from your foot all the way to the brain; from there, nerve signals travel back to the leg muscles and cause them to contract, drawing back the foot. Dozens of neurons can be involved in such a circuit, necessitating a sophisticated communication system to rapidly convey signals between cells. Also, because individual neurons can be up to 3 feet long, a rapid-relay mechanism within the neurons themselves is required to transmit each signal from the site where it is received to the site where it is passed on to a neighboring cell. Two mechanisms have evolved to transmit nerve signals. First, within cells, electrical signals are conveyed along the cell membrane. Second, for communication between cells, the electrical signals generally are converted into chemical signals conveyed by small messenger molecules called neurotransmitters.

## Signal Transmission Within Nerve Cells

The mechanism underlying signal transmission within neurons is based on voltage differences (i.e., potentials) that exist between the inside and the outside of the cell. This membrane potential is created by the uneven distribution of electrically charged particles, or ions, the most important of which are sodium (Na^+^), potassium (K^+^), chloride (Cl^−^), and calcium (Ca^2+^). Ions enter and exit the cell through specific protein channels in the cell’s membrane. The channels “open” or “close” in response to neurotransmitters or to changes in the cell’s membrane potential. The resulting redistribution of electric charge may alter the voltage difference across the membrane. A decrease in the voltage difference is called depolarization. If depolarization exceeds a certain threshold, an impulse (i.e., action potential) will travel along the neuron. Various mechanisms ensure that the action potential propagates in only one direction, toward the axon tip. The generation of an action potential is sometimes referred to as “firing.”

**Figure:**
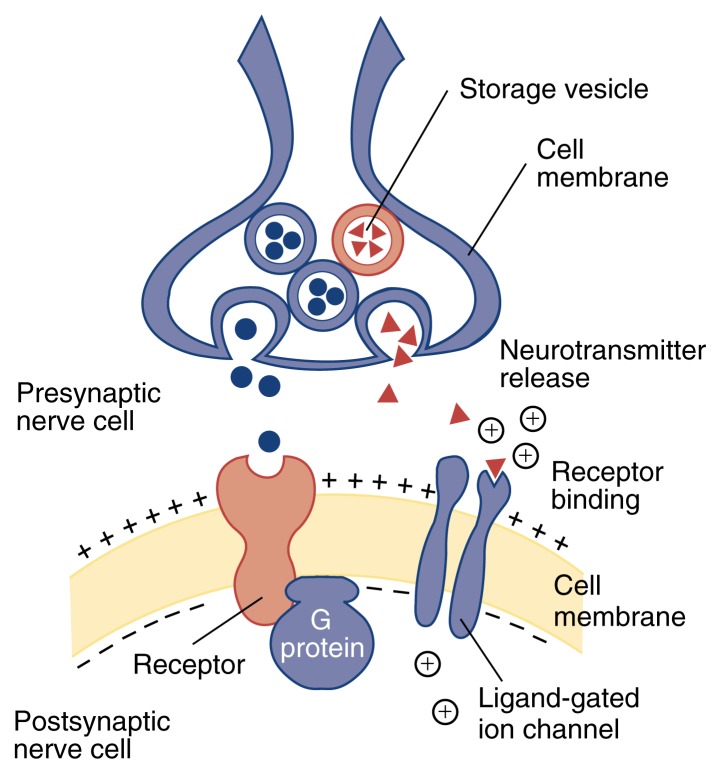
Signal transmission across the synaptic cleft. The binding of neurotransmitters (shown as triangles) to receptors that act as ligand-gated ion channels causes these channels to open, leading in some cases to a depolarization of the part of the membrane closest to the channel. Depolarization results in the opening of other ion channels, which in turn may generate an action potential. Neurotransmitters (shown as circles) that bind to second messenger-linked receptors initiate a complex cascade of chemical events that can produce changes in cell function. In this schematic, the first component of such a signaling cascade is a G protein.

### Signal Transmission Between Cells

Communication among neurons typically occurs across microscopic gaps called synaptic clefts. Each neuron may communicate with hundreds of thousands of other neurons. A neuron sending a signal (i.e., a presynaptic neuron) releases a chemical called a neurotransmitter, which binds to a receptor on the surface of the receiving (i.e., postsynaptic) neuron. Neurotransmitters are released from presynaptic terminals, which may branch to communicate with several postsynaptic neurons. Dendrites are specialized to receive neuronal signals, although receptors may be located elsewhere on the cell. Approximately 100 different neurotransmitters exist. Each neuron produces and releases only one or a few types of neurotransmitters, but can carry receptors on its surface for several types of neurotransmitters.

To cross the synaptic cleft, the cell’s electrical message must be converted into a chemical one. This conversion takes place when an action potential arrives at the axon tip, resulting in depolarization. The depolarization causes Ca^2+^ to enter the cell. The increase in intracellular Ca^2+^ concentration triggers the release of neurotransmitter molecules into the synaptic cleft.

Two large groups of receptors exist that elicit specific responses in the receptor cell: Receptors that act as ligand-gated ion channels result in rapid but short-lived responses, whereas receptors coupled to second-messenger systems induce slower but more prolonged responses.

#### Ligand-Gated Channel Receptors

When a neurotransmitter molecule binds to a receptor that acts as a ligand-gated ion channel, a channel opens, allowing ions to flow across the membrane (see figure). The flow of positively charged ions into the cell depolarizes the portion of the membrane nearest the channel. Because this situation is favorable to the subsequent generation of an action potential, ligand-gated channel receptors that are permeable to positive ions are called excitatory.

Other ligand-gated channels are permeable to negatively charged ions. An increase of negative charge within the cell makes it more difficult to excite the cell and induce an action potential. Such channels accordingly are called inhibitory.

#### Second Messenger-Linked Receptors

Second messengers (e.g., G proteins) are molecules that help relay signals from the cell’s surface to its interior. Neurotransmitters that bind to second messenger-linked receptors, such as dopamine, initiate a complex cascade of chemical events that can either excite or inhibit further electrical signals (see figure). The neurotransmitters also may attach to receptors on the transmitting cell’s own presynaptic sites, beginning a feedback process that can affect future communication through that synaptic cleft.

With so many different receptors on its cell surface, some of the signals the neuron receives will have excitatory effects, whereas others will be inhibitory. In addition, some of the signals (e.g., those transmitted through ligand-gated channels) will induce fast responses, whereas others (e.g., those transmitted through second messenger-linked proteins) will trigger slow responses. The integration by the neuron of these often conflicting signals determines whether the neuron will generate an action potential, release neurotransmitters, and thereby exert an influence on other neurons.

## Neurotransmitters and Alcohol

Among the neurotransmitters of most interest to alcohol researchers are dopamine, serotonin, glutamate, gamma-aminobutyric acid (GABA), opioid peptides, and adenosine, all of which are featured in this special section. These molecules generally fall into three categories: (1) excitatory neurotransmitters (e.g., glutamate), which activate the postsynaptic cell; (2) inhibitory neurotransmitters (e.g., GABA), which depress the activity of the postsynaptic cell; and (3) neuromodulators (e.g., adenosine), which modify the postsynaptic cell’s response to other neurotransmitters. Neurons that release these substances form the basis of neural circuits that link different areas of the brain in a complex network of pathways and feedback loops. The integrated activity of these circuits regulates mood, activity, and the behaviors that may underlie disorders such as alcoholism.

